# Estrogen regulation in the prostate underlies racial disparity in men with benign prostatic hyperplasia

**DOI:** 10.1002/path.70000

**Published:** 2025-11-29

**Authors:** Teresa T Liu, Laura E Pascal, Emily A Ricke, Ana Lucila Bautista‐Ruiz, Justin Townsend, Glenn O Allen, Rajiv Dhir, Douglas W Strand, Donald B DeFranco, William A Ricke

**Affiliations:** ^1^ Wegmans School of Pharmacy, St. John Fisher University Rochester NY USA; ^2^ Department of Urology, George M. O'Brien Center of Research Excellence University of Wisconsin–Madison Madison WI USA; ^3^ Department of Pharmacology and Chemical Biology University of Pittsburgh Pittsburgh PA USA; ^4^ UPMC Hillman Cancer Center University of Pittsburgh School of Medicine Pittsburgh PA USA; ^5^ College of Osteopathic Medicine Touro University Middletown NY USA; ^6^ Department of Pathology University of Pittsburgh Medical Center Pittsburgh PA USA; ^7^ Department of Urology University of Texas Southwestern Dallas TX USA

**Keywords:** BPH, racial disparity, estrogen signaling, multiplex IHC, ERβ signaling, normal prostate, LUTS

## Abstract

Lower urinary tract symptoms (LUTS), associated with benign prostatic hyperplasia (BPH), are an aging‐related disease, with more than 210 million cases worldwide. Estrogen exposure and estrogen regulation have been implicated in a variety of disease processes, with estrogen receptor (ER)‐α pathways associated with disease progression and ERβ pathways considered to be disease‐protective through enhanced apoptosis and reduced cellular proliferation. Preclinical models of LUTS/BPH have shown that ERα activation contributes to disease initiation and progression. Self‐identified African American (AA) men have a high incidence of LUTS/BPH, with increased incidence of non‐surgical treatment failure, larger prostates at time of surgery, and surgery occurring at a younger age compared with self‐identified European American (EA) men. While circulating estrogen levels are higher in AA individuals, regulation of ERs, particularly ERβ, in normal and LUTS/BPH human prostate has not been well characterized. In this study, we examined differences in ER expression between peripheral zone (PZ) and transition zone (TZ) prostate tissues using multiplex, multispectral imaging. Additionally, we assessed changes in ERs and steroid metabolism genes involved in ERβ signaling between normal and LUTS/BPH prostate samples. Our study revealed underlying differences in steroid metabolism gene expression between normal AA and EA prostates, which were further altered with LUTS/BPH. Importantly, the contribution of ERα to LUTS/BPH was more pronounced in EA prostate samples, whereas AA prostate samples exhibited an overall increase in the expression of both ER and estrogen metabolism‐related genes. Although estrogens have also been implicated in collagen deposition in the prostate of LUTS/BPH patients, we did not observe significant differences in collagen deposition between AA and EA samples. These results suggest that racial differences in steroid hormone signaling pathways within the benign prostate represent a promising area for the development of precision‐based therapies to reduce LUTS in aging men. © 2025 The Author(s). *The Journal of Pathology* published by John Wiley & Sons Ltd on behalf of The Pathological Society of Great Britain and Ireland.

## Introduction

Benign prostatic hyperplasia (BPH) is an aging‐associated disease, with more than 210 million cases worldwide; nearly all men will encounter some clinical lower urinary tract symptoms (LUTS) in their lifetime [1, 2, 3]. The development of LUTS/BPH is multifactorial but has been associated with an aging‐mediated decrease in circulating testosterone levels, coinciding with a lower testosterone‐to‐estrogen (T:E) ratio [3, 4, 5, 6, 7]. Elevated levels of 17β‐estradiol (E2) have been shown to increase the risk of stroke and cardiovascular disease in aging men [8, 9, 10]. Furthermore, men with rheumatoid arthritis exhibit higher levels of E2 but lower levels of dehydroepiandrosterone (DHEA), the major circulating testosterone metabolite, compared with men without chronic inflammation [11].

Both major forms of the estrogen receptor (ER), ERα and ERβ, are expressed in the prostate, with ERα predominantly localized within stromal cells and ERβ expressed in both stromal and epithelial compartments [12, 13, 14]. Previous studies have implicated ERs in prostatic disease, with ERα associated with LUTS in humans and mice, while ERβ may limit prostatic hyperplasia through enhanced epithelial cell apoptosis [15, 16]. Additionally, *in vitro* studies using benign prostate cell lines suggest that appropriate balancing of ER signaling is important for an effective response to 5α‐reductase inhibitor (5ARI) treatment in LUTS/BPH [17].

**Figure 1:**
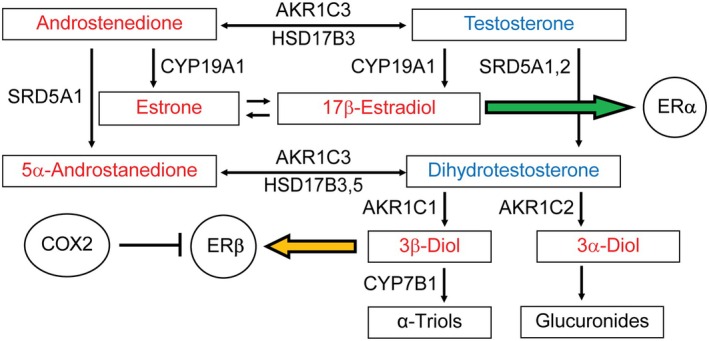
Estrogen signaling pathway within the prostate. ERα activation occurs upon binding of high‐affinity ligand 17β‐estradiol. ERβ activation occurs upon binding of high‐affinity ligand 3β‐diol. AKR1C1 and CYP7B1 are integral in the metabolism of 3β‐diol from DHT. COX2 inhibits ERβ activation through the production of reactive oxygen species. Androgens are depicted in blue; estrogens are depicted in red.

AKR1C1 is the predominant enzyme responsible for mediating dihydrotestosterone (DHT) metabolism to 3β‐androstanediol (3β‐diol), an ERβ agonist, and has been identified as a potential biomarker for prostate cancer progression (Figure 1) [18, 19, 20]. Conversely, CYP7B1 catalyzes the metabolism of 3β‐diol to the inactive metabolite 5α‐androstanetriol [21, 22]. The accumulation of 3β‐diol is influenced by COX2, a cyclooxygenase associated with prostatic inflammation [23, 24]. This suggests that an appropriate balance of sex steroid hormones is crucial for the maintenance of normal prostate size and urinary function with increasing age.

African American (AA) men have a higher incidence of BPH with increased incidence of non‐surgical treatment failure, larger prostates at time of surgery, and surgery occurring at a younger age compared with their European American (EA) counterparts [25, 26, 27]. Similar to the racial disparity in prostate cancer, this susceptibility appears independent of socioeconomic factors or access to healthcare [27, 28, 29, 30]. Additionally, LUTS/BPH in EA men does not always correlate with prostate volume, whereas prostate volume increases in AA men with age [31, 32]. Racial and ethnic differences observed in BPH incidence may therefore be due in part to genetic, cellular, and/or hormonal differences [25, 32, 33, 34, 35, 36]. Although large‐scale genome‐wide association studies in prostate cancer have identified several single nucleotide polymorphisms associated with aggressive disease, these biomarkers are inconsistent between EA and AA men [37, 38, 39]. However, several studies have shown that circulating steroid hormone levels differ between AAs and EAs; specifically, AAs exhibit higher levels of circulating estrogens independent of age [31, 33, 40, 41, 42]. These differences in estrogen steroid metabolism may lead to an increased susceptibility to LUTS/BPH, as well as an altered response to standard treatment with 5α‐reductase inhibitors (5ARIs).

In this study, we investigated the expression and localization of ERα and ERβ in prostate tissue, as well as key enzymes responsible for the accumulation of ERβ ligands (AKR1C1, CYP7B1) and ERβ receptor activation (COX2). Using these markers, we examined expression patterns in normal and BPH tissue. Additionally, we assessed how these markers affected prostate fibrosis, a known mechanism of treatment resistance.

## Materials and methods

### Patient cohort and prostate tissue acquisition

Prostate tissue samples were collected from three separate sites: University of Wisconsin–Madison (UW), University of Pittsburgh (Pitt), and University of Texas Southwestern (UTSW). Specimens from UW were collected as previously described [43]. These tissues were assembled into 6‐mm cores arranged as a tissue microarray (TMA) including benign prostate tissue (PZ; *n* = 96 cores, 48 patients) and BPH (*n* = 48 cores, 24 patients) [43, 44]. From the Pitt Biospecimen Core, normal and BPH tissues were obtained from both EA and AA patients. Race was determined through self‐identification rather than ancestry. Normal transition zone prostate tissue was collected from low‐grade, localized prostate cancer patients undergoing radical prostatectomy. BPH samples were collected from patients undergoing transurethral resection of the prostate (TURP). From the UTSW Biorepository, BPH samples were collected from EA and AA patients undergoing simple prostatectomy.

### Multiplex immunofluorescence staining

Tissues for immunohistochemistry (IHC) were fixed in 10% normal buffered formalin (NBF) and paraffin‐embedded. In brief, 5‐μm sections were cleared and rehydrated. Serial slides were stained with hematoxylin and eosin (H&E), and BPH nodules and normal adjacent prostate (NAP) were identified by a board‐certified GU (genitourinary) pathologist (RD). Multiplex immunofluorescence was performed according to OPAL manufacturer protocols (Akoya Biosciences, Marlborough, MA, USA). In brief, antigen retrieval was performed using a Decloaking Chamber™ (Biocare Medical, Pacheco, CA, USA). Initial antigen retrieval was performed for 30 min at 110 °C, while each subsequent antigen retrieval step to remove previous primary and secondary antibodies was performed for 5 min at 110 °C. Tris‐EDTA, pH 9.0, was used in staining for nuclear proteins, and 10 mm citrate buffer, pH 6.0, was used for cytoplasmic proteins. Sections were stained with anti‐AKR1C1 (1:100; PA5‐21672; Thermo Fisher Scientific, Waltham, MA, USA), anti‐COX2 (1:600; CRM306A, BioCare Medical), anti‐CYP7B1 (1:100; PA5‐28121, Thermo Fisher Scientific), anti‐E‐cadherin (1:400; #3195; Cell Signaling Technology, Danvers, MA, USA), anti‐ERα (1:100; IHC00006; Bethyl Laboratories, Montgomery, TX, USA), and anti‐ERβ (1:500; BS2429; BioWorld Technology, Bloomington, MN, USA). Each antibody was optimized for appropriate fluorophores and sequence for optimal staining and visualization (Table 1).

**Table 1:** Multispectral multiplex IHC protein targets.

Antibody	Catalog No.	Source	AR buffer	OPAL fluorophore
AKR1C1	PA5‐21672	Thermo Fisher Scientific, Waltham, MA, USA	Citrate	620
COX2	CRM306A	BioCare Medical, Pacheco, CA, USA	Citrate	540
CYP7B1	PA5‐28121	Thermo Fisher Scientific	Citrate	650
E‐cadherin	#3195	Cell Signaling, Danvers, MA, USA	Citrate	690
ERα	IHC00006	Bethyl Laboratories, Montgomery, TX, USA	Tris‐EDTA	520
ERβ	B52429	BioWorld Technology, Bloomington, MN, USA	Tris‐EDTA	570

### Multiplex image acquisition, deconvolution, and quantification

For each patient sample, at least three non‐overlapping images were acquired for each unique region (i.e. BPH, NAP, stromal, and luminal). Images were acquired using the Vectra 2 Scope System (PerkinElmer, Waltham, MA, USA) and quantified using InForm software (Akoya Biosciences). An unstained prostate section was processed simultaneously with multiplex samples and imaged to quantify autofluorescence. Each fluorophore was spectrally unmixed using the synthetic stain libraries available through the InForm software (Akoya Biosciences). Cell and tissue segmentation was performed to examine protein localization. Fluorescence counts were quantified for each antibody to determine the relative expression of each protein, with brighter pixels indicating higher relative expression. The counts were then normalized for exposure across all images, taking into account gain, bit depth, well depth, and exposure time. The integrated density was calculated by (counts × relative well depth)/(2^bit depth^ × exposure time × gain × binning area).

### Picrosirius red staining and collagen quantification

Picrosirius red (PSR) staining was performed following the manufacturer's protocol. Images of the relevant prostate compartments were captured at 20× magnification under both brightfield and circular polarized filters. Images were normalized and quantified as previously described using ImageJ [45].

### Statistical analyses

Statistical significance for comparing normal samples was assessed using one‐way ANOVA with Tukey's *post‐hoc* analysis. Statistical significance assessing changes based on either race or disease was calculated using Student's *t*‐test. Statistical significance for assessing non‐parametric changes based on both race and disease was calculated using Kruskal–Wallis two‐way ANOVA.

## Results

### Tissue identification and segmentation

**Figure 2:**
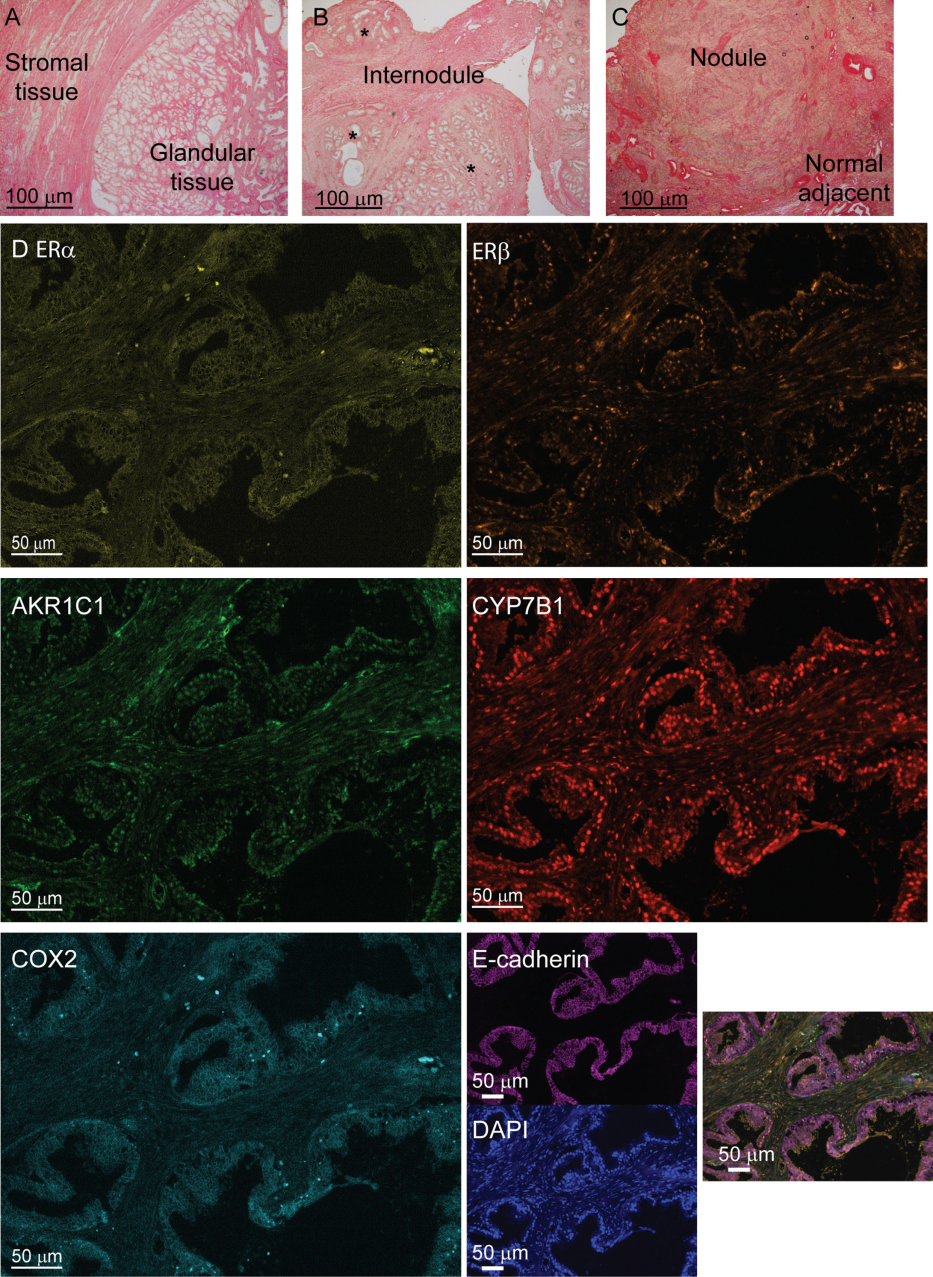
Tissue and cellular segmentation of multiplex IHC prostate tissue. (A) Representative brightfield image of the normal transition zone (TZ) shows both glandular and stromal tissue. (B) Representative image of prostate from a benign prostatic hyperplasia (BPH) patient shows classification of glandular nodules surrounding internodule (IN) tissue. * indicates prostate nodules. (C) Representative image of BPH tissue identifying a nodule next to normal adjacent tissue. (D) Deconvolution of each fluorophore using InForm allows for the visualization of individual proteins within each tissue.

For normal samples, glandular and stromal compartments were identified in each sample (Figure 2A). In BPH tissue sections, nodules, internodules (IN), and NAP were identified, where possible. IN were defined as normal tissue adjacent to more than one nodule (Figure 2B). NAP was characterized by normal tissue adjacent to a single nodule (Figure 2C). Similar tissue compartments and structures were identified within both AA and EA prostate samples (Table 2). Multiplex fluorescence IHC was performed on the tissues to examine the expression of six proteins within one tissue section. Each protein was stained with a specific fluorophore and could be visualized together as one image that could be deconvoluted to a specific spectrum (supplementary material, Figure S1A). All images were segmented into tissue compartments consisting of stroma (green), epithelium (red), and not tissue (blue; supplementary material, Figure S1B). Each image was also segmented into cells based on DAPI nuclear staining (supplementary material, Figure S1C). This staining and InForm software allowed for the examination of multiple proteins in one tissue section, as well as for assessing protein localization (Figure 2D).

**Table 2:** Distribution of prostate tissue compartments and structures.

	European American (EA)	African American (AA)
**Normal**	**10**	**11**
Glandular	10	9
Stromal	4	11
**BPH**	**15**	**22**
Normal adjacent glandular	10	14
Normal adjacent stromal	6	15
Internodule	13	15
Glandular nodule	14	16

### Differential expression of estrogen signaling pathway proteins between peripheral and transitional zone prostate

**Figure 3:**
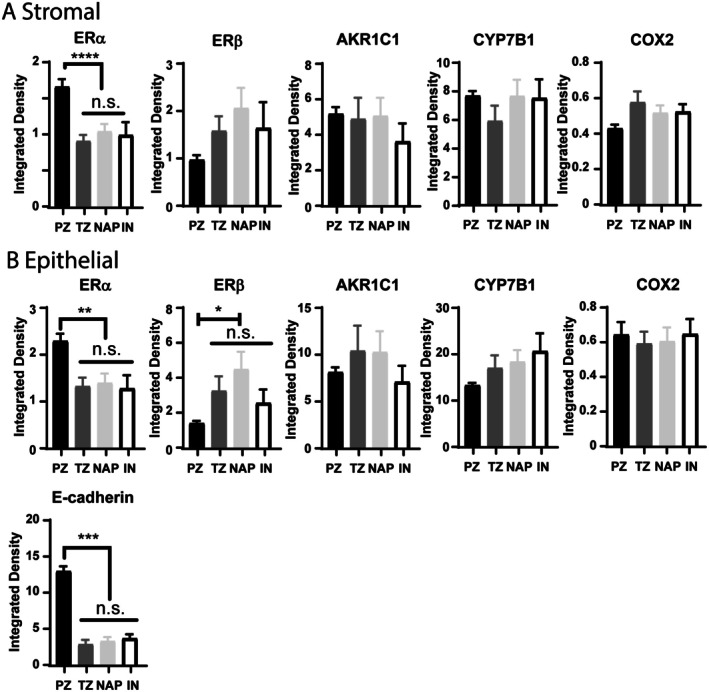
Estrogen signaling pathway in normal tissue differs within different zones of the prostate. (A) Stromal expression of ERα is unchanged within the transition zone (TZ), normal adjacent prostate (NAP), and internodules (IN). The expression of ERα is significantly lower than that for prostate originating from the peripheral zone (PZ). There were no significant differences in stromal ERβ, AKR1C1, CYP7B1, and COX2. (B) Epithelial expression of ERα, ERβ, and E‐cadherin is unchanged within the TZ, NAP, and IN. ERα and E‐cadherin were significantly increased in the PZ, while ERβ was significantly decreased. There were no significant differences in epithelial AKR1C1, CYP7B1, and COX2. *p < 0.05, **p < 0.01, ***p < 0.001, ****p < 0.0001; n.s., not significant.

In many prostate cancer studies, normal adjacent PZ and BPH tissue from the TZ are used interchangeably; in BPH studies, normal adjacent PZ is used as controls for BPH. Given the three cohorts of tissues, we had the unique opportunity to examine the differences in expression of various proteins in normal prostate tissue. The normal tissue in the TMA originated from the peripheral zone (PZ; *n* = 48), while the normal tissue from the Pittsburgh Biospecimen Core originated from the transition zone (TZ; *n* = 28). These tissues originated from patients with localized, low‐grade prostate cancer. In many of the BPH samples, we could identify NAP (*n* = 29) adjacent to a single nodule along with IN (*n* = 25) surrounded by multiple nodules. We examined each protein in the stromal compartment and saw a significant difference in ERα expression between the PZ, TZ, NAP, and IN (Figure 3A). Examining the epithelial compartment, there were significant differences in ERα, ERβ, and E‐cadherin between the PZ and the TZ, NAP, and IN (Figure 3B). Although the PZ was not significantly different in all instances, BPH originates within the TZ; thus, we removed the PZ from all downstream analysis. Additionally, since the TZ, NAP, and IN were not statistically different for any of the proteins examined, we collectively referred to these tissues as normal prostatic tissue (NPT).

### Racial differences in expression and prostate tissue localization of proteins involved in the estrogen signaling pathway

**Figure 4:**
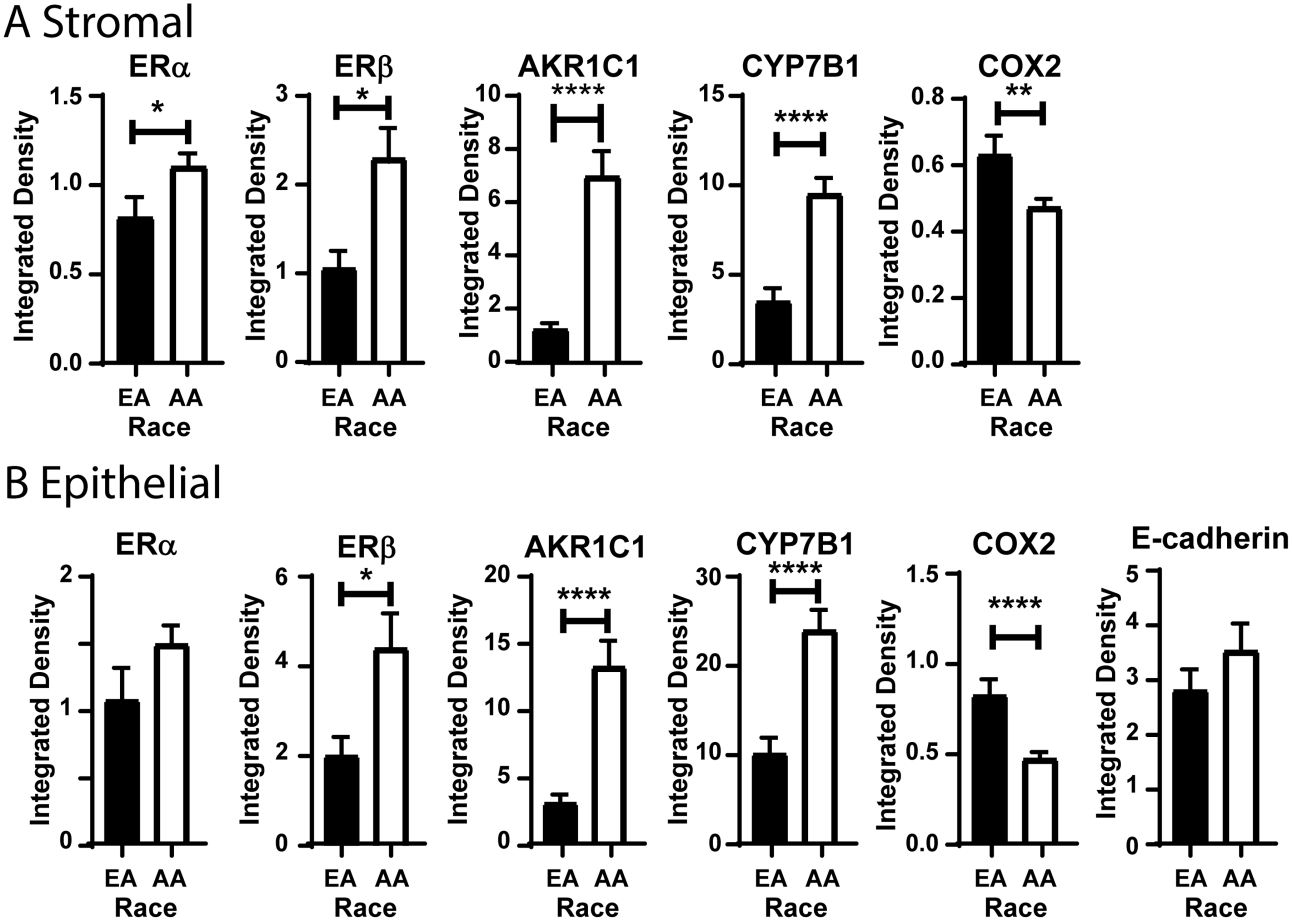
Estrogen signaling pathway is different in NPT between EA and AA men. (A) Stromal ERα, ERβ, AKR1C1, and CYP7B1 are significantly increased in African American (AA) men compared with European American (EA) men. Stromal COX2 is significantly decreased in AA men. (B) Epithelial ERβ, AKR1C1, and CYP7B1 are significantly increased in AA men compared with EA men. Epithelial COX2 is significantly decreased in AA men compared with EA men. ERα and E‐cadherin are unchanged between races. *p < 0.05, **p < 0.01, ****p < 0.0001.

Given the prevalence of prostate disease in AA men, we assessed the protein expression differences between the NPT in EA and AA men. Within the stroma of NPT, there was a significant increase in the expression of ERα, ERβ, AKR1C1, and CYP7B1 protein in AA men compared with EA men (Figure 4A). In the same tissues, there was also a significant decrease in COX2. Changes in the protein expression between AA and EA men were also recapitulated within the epithelium (Figure 4B). As expected, there were no significant differences in epithelial ERα as it is more prevalent in prostate stroma. No difference in E‐cadherin expression was observed, suggesting no difference in barrier function within the prostate.

### Estrogen signaling pathway is distinct between races and within BPH versus normal prostatic tissue

**Figure 5:**
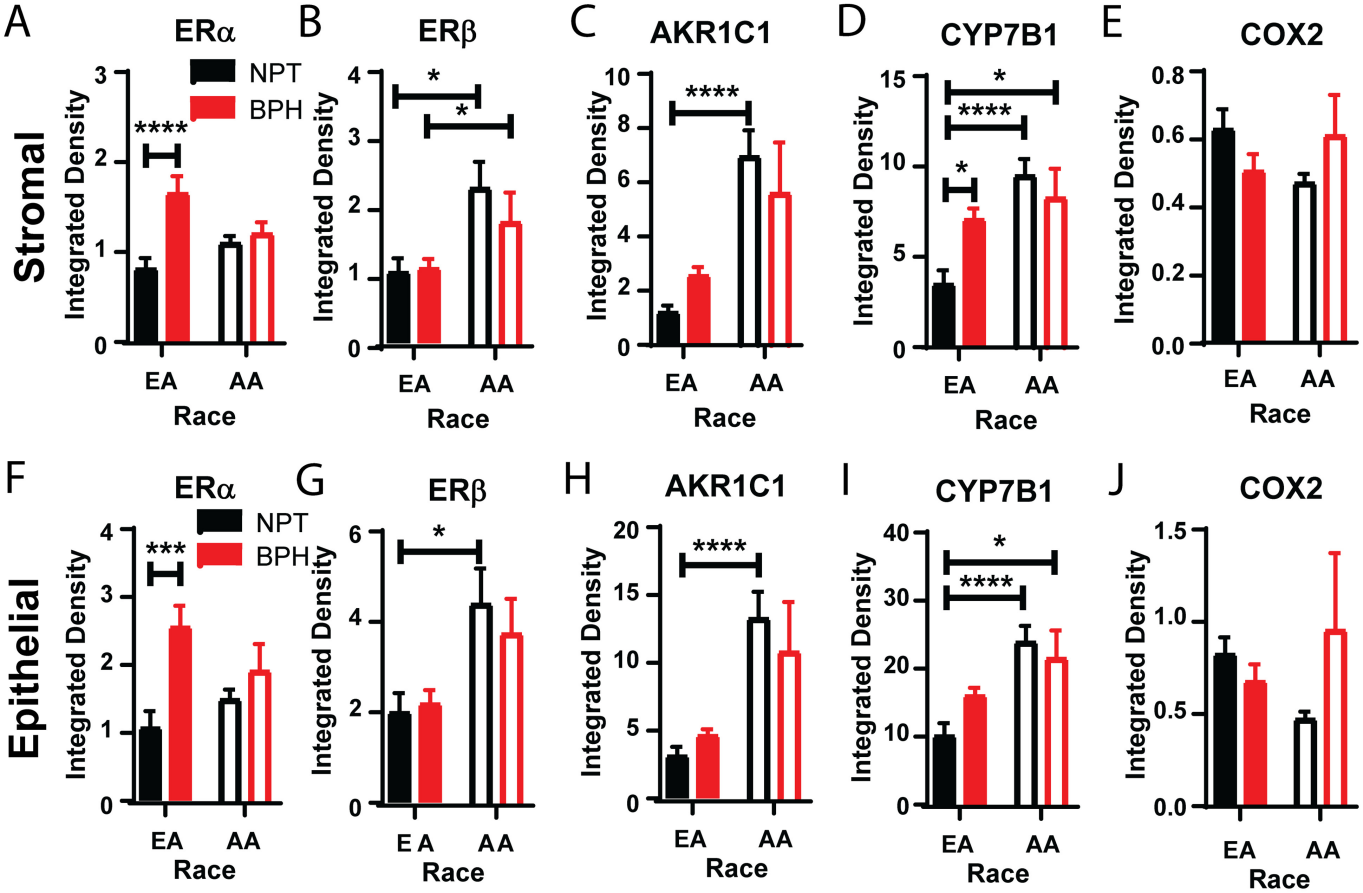
Contribution of the estrogen signaling pathway to BPH is different between races. (A) Stromal ERα is significantly increased in European American (EA) men with benign prostatic hyperplasia (BPH) compared with normal prostatic tissue (NPT). There is no significant difference in ERα expression in African American (AA) men. (B) Stromal ERβ was unchanged between NPT and BPH in EA and AA men. However, ERβ was significantly higher in AA men than in EA men in both NPT and BPH. (C) Stromal AKR1C1 was not different between NPT and BPH. AKR1C1 was significantly higher in AA NPT than in EA NPT. (D) Stromal CYP7B1 was significantly increased in BPH compared with NPT in EA men. There was no significant difference between NPT and BPH in AA men. However, AA men had significantly higher expression of stromal CYP7B1 compared with EA men. (E) Stromal COX2 was unchanged between BPH and NPT in both EA and AA men. (F) Epithelial ERα was significantly increased in BPH compared with NPT in EA men. There was no difference in AA men. (G) Epithelial ERβ showed no difference with disease in either EA or AA men. However, ERβ was significantly increased in AA NPT compared with EA NPT. (H) Epithelial AKR1C1 showed no significant difference with disease in either EA or AA men. AKR1C1 was significantly higher in AA NPT than in EA NPT. (I) Epithelial CYP7B1 was unchanged between NPT and BPH in both EA and AA men. However, AA men had significantly higher expression of epithelial CYP7B1 compared with EA men. (J) No significant difference was observed between COX2 expression between BPH and NPT in either EA or AA men. *p < 0.05, ***p < 0.001, ****p < 0.0001.

While ERα has been thought to drive prostate proliferation and fibrosis, the effectiveness of selective estrogen receptor modulators to treat LUTS/BPH has been inconsistent [46, 47]. Given the significant differences within NPT between races, we examined the expression of proteins involved in estrogen signaling in BPH samples. Stromal ERα was significantly increased in EA with LUTS/BPH compared with NPT control (Figure 5A). However, the expression of ERα was unchanged with disease in AA men. The expression of ERβ was unchanged with disease in EA and AA men (Figure 5B). However, the expression of ERβ was significantly higher in AA men than in EA men. There was also no significant difference in AKR1C1 expression with disease in either cohort of men (Figure 5C). Expression of CYP7B1 was significantly increased in EA with BPH, with no difference in AA with disease (Figure 5D). Interestingly, CYP7B1 expression was significantly increased in AA men with and without BPH compared with EA NPT. Expression of COX2 was unchanged between AA and EA men regardless of disease status (Figure 5E). Within the prostate epithelium, ERα was also significantly increased in EA men with BPH, with no difference in expression in AA men (Figure 5F). ERβ and AKR1C1 expression was only significantly different in NPT between EA and AA men, with no significant difference with BPH (Figure 5G,H). CYP7B1 expression in the epithelium was not significantly different with disease, but AA men had higher expression compared with EA men regardless of disease status (Figure 5I). Similar to the stroma, COX2 expression remained unchanged (Figure 5J).

### Collagen accumulation is decreased in BPH nodule irrespective of race

**Figure 6:**
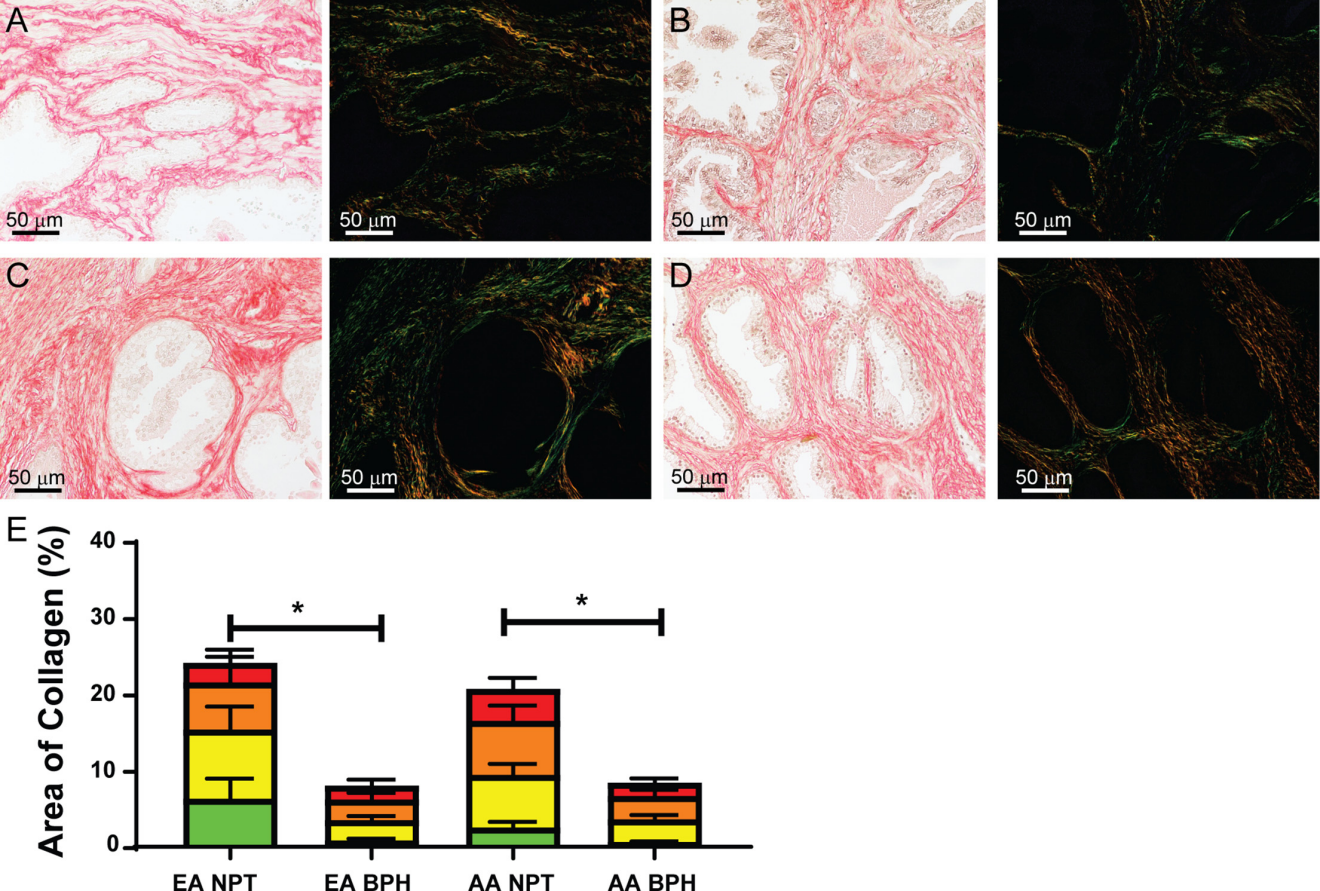
PSR staining assesses total collagen deposition and collagen fiber thickness. (A–D) Representative images of normal prostatic tissue (NPT) from European American (EA) men (A), benign prostatic hyperplasia (BPH) from EA men (B), NPT from African American (AA) men (C), and BPH from AA men (D), with brightfield (left) and birefringence (right). (E) Quantification of birefringent images shows a significant decrease of fibrosis within the BPH nodule but no difference in collagen thickness distribution. Collagen bundle thickness under circular polarized light ranged from green (thinnest) to yellow to orange to red (thickest). *p < 0.05.

Fibrosis has been linked to LUTS/BPH disease progression and treatment resistance [48, 49]. The accumulation of collagen has also been associated with a change in estrogen signaling [50]. Collagen deposition was decreased in the glandular nodules of BPH compared with the PZ normal tissues from the UW–Madison cohort [51]. Collagen bundles were assessed in EA NPT using PSR staining (Figure 6A) in EA BPH (Figure 6B), AA NPT (Figure 6C), and AA BPH (Figure 6D). There was a significant decrease in total collagen in BPH samples compared with NPT in both EA and AA men (Figure 6E). However, the distribution of collagen fiber thickness remained the same between normal and BPH. Additionally, there was no significant difference in total collagen deposition between EA and AA men. Quantification of birefringence showed collagen bundle thickness from thinnest (green) to thickest (red). The distribution of collagen bundle thicknesses was similarly represented in all groups.

## Discussion

The findings of this study suggest altered regulation of estrogen signaling in prostate tissue, associated with the benign prostatic disease. Differences in the components of the ERα and ERβ signaling pathway in AA and EA men suggest an underlying biological mechanism for racial differences outside of socioeconomic factors in disease progression and treatment efficacy. While this study classified patients based on self‐identified race, the significant differences between the groups provide valuable insight into potential targetable mechanisms for the treatment of LUTS/BPH.

Most studies within the prostate focus on malignant disease, either using TZ prostate tissue from surgical BPH patients or PZ prostate tissue from low‐grade prostate cancer patients undergoing radical prostatectomy [52, 53]. In the analysis of LUTS/BPH, obtaining appropriate age‐matched normal control tissues proved challenging, as previous studies have shown that some histopathological changes characteristic of BPH occur in the prostate with aging in all men, irrespective of clinical symptoms [54, 55]. The current study demonstrates that appropriate normal controls need to be carefully considered. While some steroid metabolism genes were unchanged between the PZ and TZ, the expression of both major ERs was significantly different between these two zones of the human prostate.

In prostate cancer, previous studies have shown a field effect in normal adjacent prostate tissue based on distance from the tumor foci [56, 57]. Interestingly, while there was a zone effect regarding the expression of ERα and ERβ, no field effect was observed between LUTS/BPH patients and normal controls. TZ tissues were derived from men without a LUTS/BPH diagnosis, while NAP and IN tissues were obtained from patients undergoing surgical interventions. There were no differences in expression between normal and disease, nor between NAP (next to only one nodule) and IN (surrounded by at least two nodules). These data highlight the importance of examining both normal and normal adjacent prostate tissue for specific proteins of interest, as local field effects may occur around the prostate nodule.

While normal prostate development and maintenance are primarily regulated by androgens, several studies have demonstrated that estrogen signaling can play an important role in maintaining normal tissue or as a driver of disease pathogenesis [58, 59, 60]. In NPT, we observed a significant increase in some components of the estrogen signaling pathway, including both ERα and ERβ, in AA compared with EA men. This suggests an underlying difference in steroid hormone metabolism and steroid receptor signaling between these groups. AKR1C1 expression was significantly increased in AA male prostate with a corresponding decrease in COX2 expression, suggesting an overactivation of ERβ. While ERβ activation is often associated with a protective phenotype, the significant increase in CYP7B1 in the AA male prostate suggests an increase in the metabolism of ERβ agonists, potentially altering the ERα:ERβ signaling homeostasis. Additionally, AA men exhibited significantly higher ERα expression within the prostate stroma, further changing the ratio of ERα:ERβ, which may underlie the predisposition of AA men to developing prostate disease and LUTS/BPH.

Given the underlying differences within the normal prostate, the contribution of estrogens to the development of disease may differ between EA and AA men. Similar to findings from mouse models, we observed a significant increase in ERα expression in EA men suggesting that ERα may be driving disease progression [16, 44]. This effect is further exacerbated by a significant increase in CYP7B1, suggesting a reduction in 3β‐adiol accumulation, even without a significant change in ERβ receptor expression. These changes are consistent with mouse models of LUTS/BPH and other disease processes centered around ER regulation [16, 23, 61].

Given these differences in EA men, therapeutic interventions that inhibit ERα signaling, such as tamoxifen or raloxifene, or augment ERβ signaling, such as Erteberel, might be predicted to exhibit therapeutic benefit. However, clinical trials assessing these interventions have not demonstrated significant improvements in LUTS [46, 62]. When we assessed the expression of these markers based on race, we observed significant differences between AA and EA men. Instead of significant alterations in these markers with disease, AA men had significant alterations at baseline in normal prostate tissue that persisted into LUTS/BPH. These baseline differences under normal conditions could explain the racial differences in disease severity and treatment resistance. Further studies examining the differences in CYP19A1 (aromatase) expression between EA and AA men could clarify the baseline differences in estrogen signaling. Since CYP19A1 metabolizes testosterone into 17β‐estradial, a high‐affinity ligand for ERα, this could give more insight into the altered estrogen signaling pathway. Additionally, examination of androgen receptor (AR) expression may elucidate the impact of alterations in steroid hormone metabolism on AR signaling differences between EA and AA men.

Prostatic fibrosis has been shown to drive treatment resistance which may be mediated through altered estrogen signaling [49, 63, 64, 65, 66, 67]. Specifically, periurethral fibrosis has been shown to be increased upon treatment resistance [68, 69]. Although this study did not examine periurethral fibrosis, quantification of total collagen showed a significant decrease in prostate fibrosis within BPH nodules compared with NPT controls in both EA and AA men. This finding is consistent with previous studies showing reduced fibrosis in and around BPH nodules [51]. Importantly, no differences in collagen accumulation were observed between AA and EA men, despite altered estrogen signaling components. In normal AA prostate tissue, there was a significant increase in estrogen signaling proteins, yet there was no significant increase in fibrosis in AA compared with EA normal prostate. Additionally, AA men with LUTS/BPH exhibited significantly decreased fibrosis. While our cohort of patients did include stromal nodules, the sample size was insufficient to detect any differences between EA and AA men. Therefore, while these data suggest a secondary mechanism by which collagen accumulates around BPH nodules, more studies are needed to assess potential racial differences in collagen accumulation around the urethra and in stromal nodules.

While this study examined several markers in the estrogen pathway in AA and EA men, there are some limitations. Firstly, all tissues were categorized based on self‐identification of race. To better understand the underlying mechanisms controlling the estrogen pathways, future studies should include larger sample sizes with more precise characterization of racial ancestry [70, 71]. Secondly, incorporating clinical data would allow for better disease stratification and treatment. While the tissues were from age‐matched patients, other clinical information [e.g. BMI, circulating serum levels, American Urological Association Symptom Index (AUASI)] would have allowed for a richer analysis. Finally, this study did not examine the underlying cause of the baseline differences between AA and EA men. The Developmental Origins of Health and Disease (DOHaD) hypothesis suggests that early‐term exposure to endocrine disrupting chemicals (EDC) can result in long‐term disease susceptibility later in life [72, 73]. Estrogen exposure has been shown to alter the epigenetic landscape of genes, thereby altering steroid homeostasis in AA men [74, 75].

Overall, these findings underscore the need for further studies into how racial differences in steroid hormone signaling between EA and AA men influence the progression, severity, and therapeutic responsiveness to selective estrogen receptor modulators of LUTS/BPH.

## Author contributions statement

TTL, LEP, DBD and WAR designed the overall study. DWS and RD assessed patient prostate samples and provided samples for the study. TTL, JT, ALB‐R and EAR performed staining, imaging, and quantitation. GOA supervised all statistical analyses within the study. TTL and LEP analyzed the data and wrote the manuscript with assistance from DBD and WAR. All authors reviewed and edited the manuscript.

## Supporting information


**Figure S1.** Cellular segmentation of multiplex IHC prostate tissue

## Data Availability

The data and images that support the findings of this study are available from the corresponding author upon reasonable request.
