# Supplementary material for: Estrogen regulation in the prostate underlies racial disparity in men with benign prostatic hyperplasia
Source: J Pathol. 2025 Nov 29;268(2):176–87. doi: 10.1002/path.70000 (PMC12805628; doi:10.1002/path.70000)
Supplement: Supplementary file 1 — Figure S1. Cellular segmentation of multiplex IHC prostate tissue [file PATH-268-176-s001.docx]

**Estrogen regulation in the prostate underlies racial disparity in men with benign prostatic hyperplasia**

TT Liu, LE Pascal *et al. J Pathol* <https://doi.org/10.1002/path.70000>

**Supplementary Figure S1**

**
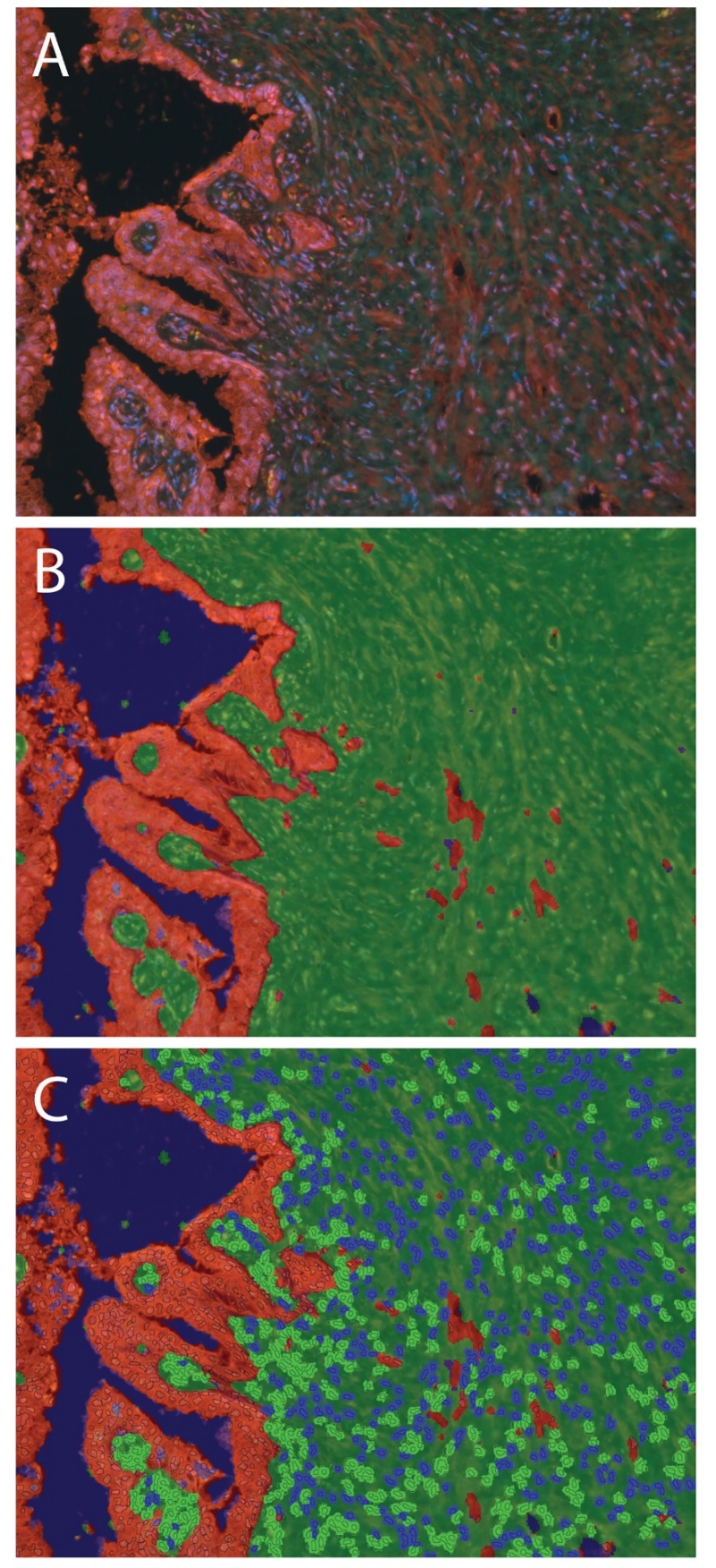
**

**Figure S1. Cellular segmentation of multiplex IHC prostate tissue.** (A) Prostate tissue stained with all six proteins without spectral unmixing. (B) Tissue segmentation using InForm using machine learning differentiates between stromal and epithelial tissue. (C) Cell segmentation using InForm classifies the cellular components (nuclei, cytoplasm) within each cell.
